# Unmasking Isolated Glucocorticoid Deficiency: Clinical Insights From 2 Cases

**DOI:** 10.1210/jcemcr/luaf316

**Published:** 2026-01-14

**Authors:** Ayushi Singhal, Jayakrishnan C Menon, Subhash Chandra Yadav, Ayush Gupta, Gunna Sriharsha, Eesh Bhatia

**Affiliations:** Department of Endocrinology, Sanjay Gandhi Postgraduate Institute of Medical Sciences, Lucknow, Uttar Pradesh 226014, India; Department of Endocrinology, Sanjay Gandhi Postgraduate Institute of Medical Sciences, Lucknow, Uttar Pradesh 226014, India; Department of Endocrinology, Jubilee Mission Medical College and Research Institute, Thrissur, Kerala 680005, India; Department of Endocrinology, Sanjay Gandhi Postgraduate Institute of Medical Sciences, Lucknow, Uttar Pradesh 226014, India; Department of Endocrinology, Sanjay Gandhi Postgraduate Institute of Medical Sciences, Lucknow, Uttar Pradesh 226014, India; Department of Endocrinology, Hormone India Diabetes and Endocrine Clinic, Visakhapatnam, Andhra Pradesh 530002, India; Department of Endocrinology, Sanjay Gandhi Postgraduate Institute of Medical Sciences, Lucknow, Uttar Pradesh 226014, India; Department of Endocrinology, Apollo Medics Superspeciality Hospital, Lucknow, Uttar Pradesh 226012, India

**Keywords:** isolated glucocorticoid deficiency, familial adrenal insufficiency, *MC2R* (melanocortin 2 receptor) gene mutation, *CYP11A1* (cytochrome P450 side-chain cleavage enzyme) gene mutation, adrenocorticotropin resistance, monogenic adrenal disorders

## Abstract

Familial glucocorticoid deficiency (FGD) is a rare autosomal recessive disorder characterized by unresponsiveness to adrenocorticotropin (ACTH) with preserved mineralocorticoid secretion. We describe 2 patients who presented with FGD. The first patient was born to a third-degree consanguineous marriage, and suffered from global developmental delay and recurrent seizures since childhood. He presented to us at age 22 years with severe hyponatremia. Laboratory investigations showed subclinical hypothyroidism, low cortisol, high ACTH, and normal plasma renin activity. After exclusion of common causes of primary adrenal insufficiency (PAI), we undertook whole-exome sequencing (WES) that revealed 2 variants—a hemizygous deletion involving exons 10 to 21 of the *AFF2* gene known to cause X-linked intellectual developmental disorder-109 and a biallelic variant in the melanocortin 2 receptor gene (NM_000529.2: c.437G > A; p.Arg146His). The second patient presented at age 25 years with severe hyponatremia and seizures. Investigations revealed isolated glucocorticoid deficiency, and WES yielded compound heterozygous variants in the *CYP11A1* gene (c.940G > A; p.Glu314Lys and c.359G > A; p.Arg120Gln). Both patients were put on glucocorticoid replacement and are doing well on follow-up. FGD should be suspected in young individuals with PAI and can be caused by a spectrum of genetic abnormalities.

## Introduction

Autoimmune adrenalitis accounts for the majority of cases of primary adrenal insufficiency (PAI) in Western countries, and infections such as tuberculosis and HIV cause most of the cases in countries where they are common [1]. Among children, adolescents, and young adults, monogenic forms of PAI are also frequently encountered, and their detection can have important implications for therapy and genetic counseling. The most common monogenic form of PAI is classic congenital adrenal hyperplasia due to *CYP21A2* mutations, followed by X-linked adrenoleukodystrophy [1].

Familial glucocorticoid deficiency (FGD) encompasses a spectrum of recessive disorders that solely affect adrenocorticotropin (ACTH) signaling and hence spares mineralocorticoid production [2]. Mutations in the melanocortin 2 receptor (*MC2R*) gene account for approximately 25% of cases (FGD type 1; OMIM No. 202200), while mutations in the melanocortin 2 receptor accessory protein (*MRAP*) gene, essential for MC2R trafficking and function, account for about 20% (FGD type 2; OMIM No. 607398) [3]. Other genes are less frequently implicated, as summarized in Table 1. The diagnosis of FGD is of practical significance since mineralocorticoid replacement is not indicated in these cases. Given its autosomal recessive inheritance, genetic counseling is crucial for early identification of at-risk individuals and informed reproductive decisions. In this report, we describe the clinical course of 2 unrelated individuals with isolated glucocorticoid deficiency.

**Table 1.** Genes associated with familial glucocorticoid deficiency

Gene symbol	Gene name	FGD subtype	Inheritance pattern	OMIM No.	Key features
*MC2R*	Melanocortin 2 receptor	FGD type 1	Autosomal recessive	202200	Most common (25% of cases); typically presents in early childhood [3]
*MRAP*	Melanocortin 2 receptor accessory protein	FGD type 2	Autosomal recessive	607398	Accounts for 20% of cases of FGD. Often severe and presents in infancy [3]
*NNT*	Nicotinamide nucleotide transhydrogenase	FGD type 4	Autosomal recessive	614736	Associated with increased oxidative stress [2]
*TXNRD2*	Thioredoxin reductase 2	FGD type 5	Autosomal recessive	617825	Rare; mitochondrial redox pathway defect [2]
*STAR*	Steroidogenic acute regulatory protein	FGD-like	Autosomal recessive	600617*^a^*	Usually associated with lipoid congenital adrenal hyperplasia [2]
*CYP11A1*	Cytochrome P450 family 11 subfamily A member 1	FGD-like	Autosomal recessive	613743*^a^*	May present later; phenotype variable [2]
*MCM4*	Minichromosome maintenance complex component 4	FGD-like	Autosomal recessive	609981	Associated with natural killer cell deficiency [2]

Abbreviation: FGD, familial glucocorticoid deficiency.

^*a*^Some OMIM entries are shared across related adrenal insufficiency phenotypes.

## Case Presentation

### Case 1

This male patient was born of a third-degree consanguineous marriage (parents were first cousins). His perinatal period was normal and his birth weight was 2.6 kg. He had delayed milestones, speaking monosyllables by age 2 years and walking independently by age 3 years. He developed recurrent episodes of generalized tonic-clonic seizures since age 4 years. He was admitted with status epilepticus in a nearby hospital at age 8 years, where he underwent evaluation including lumbar puncture and brain imaging, and was diagnosed with encephalitis. No electrolyte abnormalities were documented. Seizures improved with antiepileptic therapy. He was admitted into a school for children with a disability at age 9 years, but was forced to drop out due to general ill health, fatigue, and poor scholastic ability. The parents give history of recurrent episodes of vomiting with frequent hospital admissions. He presented to our hospital at age 22 years with intractable seizures and severe hyponatremia, prompting referral to the endocrinology department. Examination was unremarkable except for generalized hyperpigmentation. He had normal stature (163 cm at presentation), which was also appropriate for his mid-parental height.

### Case 2

A 25-year-old man presented with recurrent episodes of dizziness, vomiting, and seizures over the past 5 years. Hyponatremia was documented on multiple occasions, with a nadir sodium of 105 mEq/L (SI: 105 mmol/L) (reference range, 135-145 mEq/L [SI: 135-145 mEq/L]). He was managed with hypertonic saline, fluid restriction, and oral salt on these occasions. Prior brain imaging and electroencephalogram were normal, and he was on brivaracetam 50 mg twice daily at presentation. He was born at term to a nonconsanguineous couple with a normal perinatal period and development. There was no history of genital ambiguity, significant past illness, or similar family history. Parents did not notice hyperpigmentation or dysphagia. On examination, height was 169 cm and weight 53 kg (body mass index, 18.6). Testicular volume was 20 mL bilaterally, pubic hair stage 5, and stretched penile length 11 cm. There was no evidence of alacrimia, and examination was otherwise unremarkable.

## Diagnostic Assessment

### Case 1

Laboratory findings at presentation are summarized in Table 2. The combination of clinical features, low serum cortisol (Am cortisol <1 µg/dL [SI: <27.6 nmol/L]; reference range, 3-25 µg/dL [SI: 83-690 nmol/L]), and elevated Am ACTH (705 pg/mL [SI: 155.3 pmol/L]; reference range, 7.3-63 pg/mL [SI: 1.6-13.9 pmol/L]) confirmed PAI. Serum DHEAS (dehydroepiandrosterone sulfate) was low (22.1 µg/dL [SI: 0.6 µmol/L]; reference range, 66.3-405.3 µg/dL [SI: 1.8-11.0 µmol/L]). He was started on glucocorticoid and mineralocorticoid replacement despite normal plasma renin activity (PRA) (1.2 ng/mL/hour [SI: 1.2 µg/L/hour]; reference range, 0.5-4.9 ng/mL/hour [SI: 0.5-4.9 µg/L/hour]). Vomiting, fatigue, seizures, and hyponatremia resolved with treatment, and his longstanding skin hyperpigmentation improved.

**Table 2.** Hormonal and biochemical parameters of the patients at presentation to our center

Laboratory tests	Patient 1	Patient 2	Normal range
Hemoglobin	13.6 gm/dL	13.0 gm/dL	12-16 gm/dL
	136 g/L	130 g/L	120-160 g/L
Total leukocyte count	10.1 × 10⁹/L	7.2 × 10⁹/L	4-11 × 10⁹/L
Platelet count	152 × 10⁹/L	200 × 10⁹/L	150-400 × 10⁹/L
Serum creatinine	1 mg/dL	0.8 mg/dL	0.5-1.6 mg/dL
	88.4 µmol/L	70.7 µmol/L	44-141 µmol/L
Serum sodium	120 mEq/L	130 mEq/L	135-145 mEq/L
	120 mmol/L	130 mmol/L	135-145 mmol/L
Serum potassium	4.5 mEq/L	3.9 mEq/L	3.5-5.5 mEq/L
	4.5 mmol/L	3.9 mmol/L	3.5-5.5 mmol/L
Blood glucose	82 mg/dL	77 mg/dL	70-100 mg/dL
	4.6 mmol/L	4.3 mmol/L	3.9-5.6 mmol/L
Serum osmolarity	ND	263 mOsmol/kg	280-300 mOsmol/kg
Urine osmolarity	ND	301 mOsmol/kg	500-800 mOsmol/kg
Urinary spot sodium	ND	30 mEq/L	20-301 mEq/L
		30 mmol/L	20-301 mmol/L
TSH	8.3 mIU/L	6.7 mIU/L	0.4-4.0 mIU/L
Total T4	ND	9.4 µg/dL	4.5-12.5 µg/dL
		120.6 nmol/L	58.0-162.0 nmol/L
Free T4	1.0 ng/dL	ND	0.9-1.9 ng/dL
	13.0 pmol/L		12-24 pmol/L
TPO antibody	30 IU/mL	21 IU/mL	<35 IU/mL
Total testosterone	340 ng/dL	384 ng/dL	250-835 ng/dL
	11.8 nmol/L	13.3 nmol/L	8.6-29.0 nmol/L
8 Am ACTH	705.0 pg/mL	1963.0 pg/mL	7.3-63.0 pg/mL
	155.3 pmol/L	432.0 pmol/L	1.6-13.9 pmol/L
11 Pm ACTH	ND	141 pg/mL	<50 pg/mL
		31 pmol/L	<11 pmol/L
Am cortisol	<1.0 µg/dL	< 0.2 µg/dL	3.0-25.0 µg/dL
	<27.6 nmol/L	<5.0 nmol/L	83.0-690.0 nmol/L
Stimulated cortisol*^a^*	ND	0.3 µg/dL	>14.5 µg/dL
		8.8 nmol/L	>400.0 nmol/L
DHEAS	22.1 µg/dL	7.4 µg/dL	66.3-405.3 µg/dL
	0.6 µmol/L	0.2 µmol/L	SI: 1.8-11.0 µmol/L
Aldosterone	ND	14.4 ng/dL	7.0-34.0 ng/dL
		0.4 nmol/L	0.2-0.9 nmol/L
PRA	1.2 ng/mL/h	0.9 ng/mL/h	0.5-4.9 ng/mL/h
	1.2 µg/L/h	0.9 µg/L/h	0.5-4.9 µg/L/h

Reference ranges may vary slightly depending on the assay used.

Abbreviations: ACTH, adrenocorticotropin; DHEAS, dehydroepiandrosterone sulfate; ND, no data; PRA, plasma renin activity; T4, thyroxine; TPO, thyroid peroxidase; TSH, thyrotropin.

^*a*^ACTH stimulation test performed using 250 µg of synthetic ACTH (cosyntropin) intramuscularly, with serum cortisol measured at baseline and 60 minutes.

Evaluation for etiology revealed no alacrimia, dysphagia, or autonomic dysfunction. Computed tomography (CT) showed atrophic adrenals. X-linked adrenoleukodystrophy was excluded by normal urinary very long-chain fatty acids. 21-Hydroxylase (21-OH) antibody estimation (using an in-house immunoprecipitation assay using in vitro transcribed and translated 21-OH [4]) was negative.

**Figure 1.**
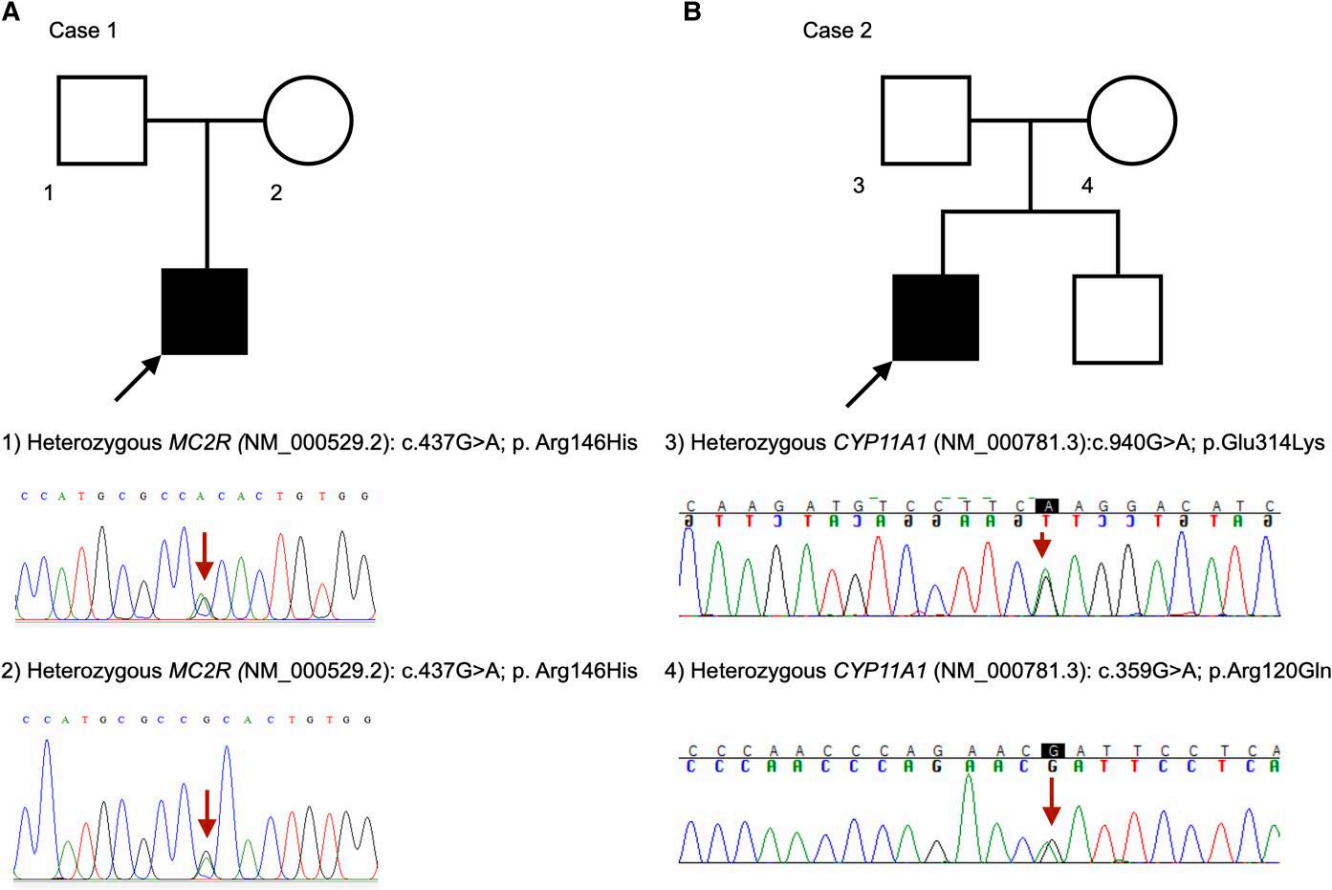
Details of family screening for genetic variants. Square—male, circle—female, black—affected, white—unaffected. A shows the pedigree chart for case 1. Sanger sequencing of parents revealed that they were heterozygous for the variant (*MC2R* [NM_000529.2]: c.437G > A; p. Arg146His). B shows the pedigree chart for case 2. The proband was compound heterozygous for 2 variants in the *CYP11A1* gene. Sanger sequencing of the parents revealed that he inherited the *CYP11A1* (NM_000781.3): c.940G > A; p. Glu314Lys variant from his father and the *CYP11A1* (NM_000781.3): c.359G > A; p. Arg120Gin variant from his mother.

Given the early age of onset and parental consanguinity, an inherited form of PAI was suspected. Exome sequencing using the Illumina HiSeq X platform (Illumina) identified 2 variants: a monoallelic deletion involving exons 10 to 21 of the *AFF2* gene, which is implicated in X-linked intellectual developmental disorder-109 (MRX109) (OMIM No. 309548) [5], and a biallelic missense variant in the *MC2R* gene (NM_000529.2: c.437G > A; p. Arg146His), previously reported in FGD1 [6-9]. The *MC2R* variant was absent in the 1000 Genomes database and had a minor allele frequency of 0.002% in gnomAD (v 4.1.0). Parental testing confirmed heterozygosity by Sanger sequencing (Fig. 1). The variant was classified as pathogenic as per American College of Medical Genetics and Genomics (ACMG) 2015 criteria (PS3 PM1 PM2 PM3 PP3 PP4) [10].

### Case 2

Laboratory investigations at presentation (see Table 2) were consistent with PAI (Am cortisol <1 µg/dL [SI: <27.6 nmol/L]; Am ACTH 1963pg/mL [SI: 432 pmol/L]). Serum DHEAS was low (7.4 µg/dL [SI: 0.2 µmol/L]). Plasma aldosterone and PRA (0.9 ng/mL/hour [SI: 0.9 µg/L/hour]) were within reference range. CT revealed bilaterally atrophic adrenals. Suspecting FGD, we performed exome sequencing using the Illumina HiSeq X platform (Illumina), which identified 2 *CYP11A1* variants in trans (confirmed by parental testing; see Fig. 1). The first variant, *CYP11A1* (NM_000781.3):c.940G > A (p.Glu314Lys) in exon 5, affects the cytochrome P450 domain, has minor allele frequencies of 0.05% in 1000 Genomes, and 0.39% in gnomAD (v4.1.0), and has been shown to cause aberrant splicing and FGD [11-14]. It was classified as “likely pathogenic” (PS3, PM3) per ACMG 2015 criteria [10]. The second variant, *CYP11A1* (NM_000781.3):c.359G > A (p.Arg120Gln) in exon 2, also lies in the cytochrome P450 domain, is extremely rare (0.001% in gnomAD; absent in 1000 Genomes), and has been previously reported in PAI [14, 15]. In silico analyses (PolyPhen-2, SIFT, MutationTaster2021) predicted it to be damaging. This variant was likewise classified as “likely pathogenic” (PM2, PM3, PP3, PP5) [10].

## Treatment

### Case 1

The genetic diagnosis of FGD1 prompted a review of the initial presentation, underscoring the relevance of normal PRA and normokalemia. Consequently, mineralocorticoid therapy was discontinued, while glucocorticoid replacement was continued.

### Case 2

The patient was started on glucocorticoid replacement at 10 mg/kg/m^2^ hydrocortisone.

## Outcome and Follow-up

Both patients have remained well over nearly 2 years of follow-up, without recurrence of seizures, vomiting, or hyponatremia. Blood pressure, aldosterone, and PRA levels have remained normal, and the families received genetic counseling.

## Discussion

We describe 2 cases of FGD with distinct genetic etiologies—one with biallelic *MC2R* mutations and the other with compound heterozygous *CYP11A1* variants. Both patients presented in their 20s with severe hyponatremia and features of glucocorticoid deficiency. Normal PRA and aldosterone levels were key clues indicating preserved mineralocorticoid function.

The first patient had FGD1, diagnosed at age 22 years, although symptoms of adrenal insufficiency were probably present earlier. His developmental delay, intellectual disability, and initial seizures were probably due to the *AFF2* gene mutation causing X-linked intellectual developmental disorder-109, as hyponatremia was not documented during the initial evaluation. This makes it difficult to determine the precise age of onset of FGD.

FGD1 typically presents later than FGD2, with the largest published cohort reporting a median age of onset of 2 years (range, 0-16 years) compared to 0.08 years (range, 0-1.6 years) for FGD2 [3]. Isolated cases with even later presentation have been described, with the oldest patient diagnosed at age 30 years [16]. This has been attributed to the severity of the mutation. Most *MRAP* mutations are splice-site or nonsense variants that abolish protein expression, resulting in complete retention of MC2R within the endoplasmic reticulum (ER). In contrast, most *MC2R* mutations are missense variants (as in our case), causing varying degrees of receptor misfolding and impaired trafficking from the ER to the cell surface, leading to reduced receptor expression (20%-100% of wild-type) and diminished ACTH signaling [9].

A feature of FGD1 that is being increasingly recognized is hypothyroidism [2]. The case presented here had subclinical hypothyroidism at presentation, while previous reports show that this can range from transient neonatal hypothyroidism to overt hypothyroidism. The exact mechanism for this has not been elucidated. Another frequently reported but variable phenotypic feature of FGD1 is tall stature, which is thought to result from the stimulatory effects of markedly elevated ACTH levels on growth plate chondrocytes [17]. This feature was not observed in our patients.

Severe loss-of-function mutations in *CYP11A1* (encoding the cholesterol side-chain cleavage enzyme, P450scc) cause primary adrenal failure with 46, XY sex reversal. However, partial inactivating mutations in *CYP11A1* have been implicated in the causation of isolated glucocorticoid deficiency [13]. While severe enzyme deficiency presents within the first few days of life, partial loss-of-function variants in *CYP11A1* are associated with later onset of PAI ranging from 6 months to 16 years [13]. Patient 2 also had mild subclinical hypothyroidism at presentation (see Table 1), which normalized after glucocorticoid replacement, suggesting a transient, nonthyroidal thyrotropin elevation. An association between thyroid disease and *CYP11A1* variants has not been reported to date. The minimal increase in serum cortisol levels following ACTH stimulation likely reflects partial preservation of enzyme activity, which may have contributed to the delayed clinical presentation.

Maharaj et al [13] undertook exome sequencing in 77 individuals or family members with PAI of unknown etiology and found that compound heterozygosity for the c.940G > A *CYP11A1* variant with a second disruptive variant accounted for 21% (16/77) of undiagnosed PAI and 4% (16/395) of all patients with adrenal insufficiency in their cohort. The majority of these patients had isolated glucocorticoid deficiency. The exact same defect was detected in our second patient, indicating that this variant is present and may account for a substantial proportion of PAI in our population as well. Maharaj and colleagues [13] demonstrated that the c.940G > A variant, which was previously predicted to be benign, caused mis-splicing, resulting in absent or dysfunctional protein.

## Learning Points

- FGD should be considered in the differential diagnosis of PAI presenting in children, adolescents, and young adults, as the age at presentation can be highly variable.
- History of consanguinity or endogamy in parents, normokalemia, and preserved mineralocorticoid function (normal aldosterone and PRA) should raise clinical suspicion for FGD.
- Early recognition of FGD is crucial, as mineralocorticoid replacement is usually unnecessary, affected individuals may exhibit characteristic phenotypic features, and genetic counseling plays an important role in family screening and risk assessment.

## Contributors

All authors made individual contributions to authorship. J.C.M., S.Y., E.B., G.S., A.S., and A.G. were involved in the diagnosis and management of the patients. J.C.M. and A.S. prepared the initial draft of the manuscript. J.C.M., S.Y., E.B., A.G., and A.S. were involved in genetic testing and interpretation. All authors reviewed and approved the final draft.

## Data Availability

Original data generated and analyzed during this study are included in this published article.

## Abbreviations

21-OH: 21-hydroxylase
ACMG: American College of Medical Genetics and Genomics
ACTH: adrenocorticotropic hormone
CT: computed tomography
DHEAS: dehydroepiandrosterone sulfate
ER: endoplasmic reticulum
FGD: familial glucocorticoid deficiency
*MC2R*: melanocortin 2 receptor gene
*MRAP*: melanocortin 2 receptor accessory protein gene
PAI: primary adrenal insufficiency
PRA: plasma renin activity
TSH: thyrotropin
WES: whole-exome sequencing
